# Decidualization Potency and Epigenetic Changes in Human Endometrial Origin Stem Cells During Propagation

**DOI:** 10.3389/fcell.2021.765265

**Published:** 2021-11-19

**Authors:** Elvina Valatkaitė, Raminta Baušytė, Aida Vitkevičienė, Diana Ramašauskaitė, Rūta Navakauskienė

**Affiliations:** ^1^ Department of Molecular Cell Biology, Institute of Biochemistry, Life Sciences Center, Vilnius University, Vilnius, Lithuania; ^2^ Centre of Obstetrics and Gynaecology of the Institute of Clinical Medicine, Faculty of Medicine, Vilnius University, Vilnius, Lithuania

**Keywords:** endometrium, menstrual blood, stem cells, decidualization, epigenetics

## Abstract

Human endometrium derived mesenchymal stem cells (hEndSCs) offer a great promise for regenerative medicine and reproductive system disorders treatment methods based on cell therapy due to their broad differentiation potential and highly efficient proliferation. In our study, we investigated the characteristics of hEndSCs that were isolated from two sources: endometrium and menstrual blood, which both contain endometrial origin stem cells. Changes in gene and protein expression levels during long-term cultivation and decidualization potential were examined in endometrial stem cells (EndSCs) and menstrual blood stem cells (MenSCs). The decidualization process was induced on early and late passages of hEndSCs using dibutyryl cyclic-AMP (db-cAMP) and medroxyprogesterone acetate (MPA) agents. We demonstrated that after long-term cultivation of hEndSCs the expression of typical mesenchymal stromal cell surface markers such as CD44, CD73, CD90, CD105 and perivascular marker CD146 remains at a similar level throughout long-term cultivation. Additionally, hematopoietic and endothelial markers CD34, CD45 were also tested, they were negative in all cases. Analyzed stem cells gene markers, such as OCT4, SOX2, NANOG, KLF4, showed similar expression in all passages of hEndSCs. RT-qPCR results demonstrated that the expression of cell cycle control associated genes - CDK2, CCNA2, CCNE2, p21, p53 and Rb, among all groups was very similar. Expression of genes associated with senescence (ATM, JUND, TOP2A, MYC) was maintained at a similar level throughout passaging. In addition, Western blot analysis was used to assess changes in proteins’ levels associated to epigenetics (EZH2, SUZ12, H3K27me3) and cell cycle control (cyclinE1, p53) during long-term cultivation. The levels of proteins associated with epigenetic changes were fluctuated slightly depending on the patient. Also, we demonstrated that in all induced hEndSCs the expression of decidualization markers Prolactin (PRL), IGFBP1 and WNT4 was upregulated. In conclusion, we demonstrated successful decidualization of stem cells derived from two reproductive system resources: endometrium and menstrual blood by using db-cAMP and MPA regardless of the length of the stem cell passaging. According these findings, we suppose that endometrium derived stem cells and menstrual blood derived stem cells could have a potency not only for endometrium tissue regeneration, but could also become a successful therapy for reproductive system disorders, including infertility or recurrent pregnancy loss.

## Introduction

Nowadays infertility is a rapidly growing global problem. The World Health Organization recognizes that infertility confers a disability, and it is now fifth on the international list of serious disabilities in women (World Health Organization. Infertility definitions and terminology. http://www.who.int/reproductivehealth/topics/infertility/definitions/en/. Access June 13, 2018). The causes of infertility can be various, although the embryo quality and the endometrial dysfunction are usually identified as the most common issues preventing successful embryo implantation and pregnancy (Unuane et al., 2011; Zhao et al., 2019). Despite quickly developing diagnostic methods for identification of specific issues causing reproductive system disorders, many women are still faced with another diagnosis—infertility of unknown origin. Of all the couples that are trying to conceive an offspring, at least 10–20% are diagnosed with infertility of unknown origin (Deroux et al., 2016; Zhao et al., 2019). Nevertheless, no clear reasons for this infertility diagnosis are determined, but it is hypothesized that one of the causes could be the thin endometrium and/or the dysfunction of endometrium. The main issue with unexplained infertility is not only frustrating diagnosis for the couple, but unfortunately, the lack of effective diagnostic and treatment methods (Siristatidis et al., 2020). Thus, there is a demand for alternative treatments such as endometrium derived stem cell and menstrual blood derived stem cell therapy (Zhao et al., 2019).

Endometrium is the inner lining layer of the female uterus. It is a dynamic tissue, which undergoes about 400 cycles of regeneration during female reproductive years and is directly connected with the outcome of blastocyst implantation and pregnancy development (Gargett and Masuda, 2010; Verdi et al., 2014). Decidualization is a crucial step in ensuring embryo attachment to the endometrial lining and this is attained by differentiation of hEndSCs to specialized decidua cells that make up endometrium (Cakmak and Taylor, 2011; Gellersen and Brosens, 2014). The regeneration of endometrium epithelial surface occurs as a consequence of endometrial mesenchymal stem cells’ proliferation and decidualization along with angiogenesis, which supplies newly formed cells with oxygen (Elsheikh et al., 2011; Zlatska et al., 2017; Cousins et al., 2018). hEndSCs are known to play a critical role in acquisition of endometrial receptivity, and thus in proper implantation of an embryo (Azizi et al., 2018). Consequently, any negative alteration associated with functionality of these cells is expected to adversely affect the pregnancy outcome. Endometrium derived mesenchymal stem cells can be obtained not only directly from endometrium, but from menstrual blood as well (Meng et al., 2007). Since endometrial stem cells (EndSCs) and menstrual blood stem cells (MenSCs) both originate from the same source—endometrium, they represent typical with mesenchymal stem cells associated properties, such as the ability to self-renew, high proliferative activity and multilineage differentiation potential. Moreover, when injected into organism, these cells show no risk of teratoma formation unlike embryonic stem cells and they have no or weak adverse immunological reactions, which makes them a promising tool for future therapies (Peron et al., 2012; Du et al., 2016; Cousins et al., 2018).

It is thought that stem cells isolated from endometrium and menstrual blood could pose a significant advancement in the treatment of reproductive system disorders, especially unexplained infertility (Hu et al., 2019). Especially, when administration of hormone therapy, surgical procedures, assisted reproductive technologies or a combination of different treatments are not efficient and other methods, for example, oocytes and/or sperm donation, surrogacy, are not acceptable for the couple (Easley IV et al., 2013; Saha et al., 2021). For these reasons, human endometrium derived mesenchymal stem cells—both EndSC and MenSC, could be a novel tool for regenerative medicine and cell therapy for treating diseases like infertility or recurrent pregnancy loss.

These cells must coordinate their differentiation patterns, proliferation and death through epigenetic regulation to maintain their applicability (Lei et al., 2014). For this reason, it is important to address epigenetic changes during long-term cultivation and evaluate the differences that may indicate about the changes in stem cells properties.

The aim of this study was to investigate the differences in gene expression levels and protein expression levels during long-term cultivation and decidualization of endometrial mesenchymal stem cells. The decidualization process was induced on early and late passages of EndSCs and MenSCs using dibutyryl cyclic-AMP (db-cAMP) and medroxyprogesterone acetate (MPA) agents and lasted 3 and 6 days. It was hypothesized that these differentiated cells may be potentially applied for endometrium tissue regeneration or for treatment of other with reproduction associated diseases. More detailed clinical trials are needed to determine the potential applicability of these cells in the clinic.

## Materials and Methods

### Isolation and Cultivation of Human Endometrium Derived Mesenchymal Stem Cells

Protocols for the study of stem cells from with patients’ specimens were approved by the Ethics Committee of Biomedical Research of Vilnius District, No. 158200-18/7-1049-550. EndSCs and MenSCs were obtained from females experiencing unexplained infertility. 12 donors were used for the experiments with endometrial stem cells and six donors for experimentations with menstrual blood stem cells. Endometrial stem cells were isolated as described previously (Tavakol et al., 2018) from scratches of the inner wall of endometrial lining and menstrual blood stem cells were obtained by collecting menstrual blood using menstrual cup. MenSCs were isolated as described by Skliute et al. (2021). Endometrium and menstrual blood mesenchymal stem cells were seeded into 25 cm^2^ plastic cell culture flasks at 1.6 × 10^**4**^ cells/cm^**2**^ density and maintained in 37°C, 5% СO_2_ incubator. The cells were cultivated in the growth medium DMEM/F12 (DMEM, Dulbecco’s Modified Eagle Medium/Nutrient Mixture F-12) (Gibco, Thermo Fisher Scientific, Waltham, MA, USA) supplemented with 10% Fetal Bovine Serum (FBS) (Gibco, Thermo Fisher Scientific, Waltham, MA, USA), 100 U/ml penicillin and 100 μg/ml streptomycin (Gibco, Thermo Fisher Scientific, Waltham, MA, USA). The medium was replaced every 2 days, and when the confluence reached 90–100%, the cells were detached from the surface using trypsin-EDTA (0.05%) solution (Gibco, Thermo Fisher Scientific, Waltham, MA, USA) and reseeded into new cell culture flasks. EndSCs and MenSCs undergo around 2–12 divisions for early passage cells (2p.–7p.) and around 28–40 for late passage cells (15p.–19p.). In gene expression and Western blot protein analysis AF-MSCs were used as a control group. AF-MSCs were isolated from five donors and cultivated according to (Savickiene et al., 2015) protocol. Cells were seeded at 1.6 × 10^4^ cells/cm^2^ density and passaged at approximately 80–90% confluence. AF-MSCs at passage three were then selected for following experiments. During cultivation of EndSCs and MenSCs we assessed proliferation and viability every time the cells were passaged. This was done by trypan blue exclusion test. The cells were trypsinized, centrifuged and resuspended in DMEM/F12 medium. Then equal volumes of cells and trypan blue 0.4% (Pharmacia LKB, Uppsala, Sweden) were mixed together and analyzed in a hemocytometer under a light microscope. The cells were counted according to their color—viable cells were transparent and dead cells were colored blue, and depending on the number of cells, viability was evaluated. EndSCs and MenSCs doubling time (DT) was assessed by monitoring cell proliferation during passaging and was calculated using formula:

$$\mathrm{DT}=\frac{\mathrm{duration}\left(\mathrm{days}\right)\times \log\left(2\right)}{\log\left(\mathrm{final concentration}\right)-\log\left(\mathrm{initial concentration}\right)}$$

### Cell Surface Markers Analysis by Flow Cytometry

For phenotypical characterization of undifferentiated EndSCs and MenSCs, cell surface markers were analyzed. For one assay around 5 × 10^4^–6 × 10^4^ cells were collected by trypsinization and centrifugation at 400 ⨯ g for 5 min. Supernatant was discarded and cells were suspended in 50 μl phosphate-buffered saline (PBS) solution (Genaxxon bioscience, Ulm, Germany) and incubated with antibodies against cell surface markers for 30 min on ice in the dark. After incubation samples were washed twice with PBS. The cells were then resuspended in 100 μl PBS and fixated with 100 μl 2% paraformaldehyde (Sigma-Aldrich, St. Louis, MO, USA) as final volume. Typical mesenchymal stem cells markers were detected using APC conjugated mouse anti-human antibodies against CD44 (Biolegend, CA, USA), CD73 (Exbio, Vestec, Czech Republic), CD90 (Exbio, Vestec, Czech Republic) and CD105 (Exbio, Vestec, Czech Republic). Markers for hematopoietic progenitor cells and endothelial cells were also analyzed using APC conjugated mouse anti-human antibodies against CD34 (Biolegend, CA, USA) and FITC conjugated mouse anti-human antibodies against CD45 (BD Pharmingen, CA, USA). Next PE-conjugated mouse anti-human antibodies against Activated Leukocyte Cell Adhesion Molecule (ALCAM) CD166 (Biolegend, CA, USA), APC conjugated mouse anti-human antibodies against c-kit stem cell factor receptor CD117 (Exbio, Vestec, Czech Republic) and against melanoma cell adhesion molecule CD146 (Biolegend, CA, USA). For isotype controls mouse IgG1-APC (Biolegend, CA, USA, AND, Exbio, Vestec, Czech Republic), IgG1-PE (Biolegend, CA, USA), IgG2a-APC (Exbio, Vestec, Czech Republic) and IgG1-FITC (Exbio, Vestec, Czech Republic) were used. Labeled samples were then measured using Partec flow cytometer (Sysmex Corporation, Cobe, Hioko, Japan) with Flowing Software 2 software.

### Decidualization Assay

Early (2p.–7p.) and late (15p.–19p.) passages of endometrial and menstrual blood mesenchymal stem cells were selected to undergo decidualization *in vitro*. Decidualization was induced by culturing cells with 0.5 μM dibutyryl cyclic-AMP (db-cAMP) (BioGems International, Inc., Westlake Village, CA, USA) and 1 μM medroxyprogesterone acetate (MPA) (Cayman Chemical Company, Ann Arbor, MI, USA) agents at 1.5 × 10^4^ cells/cm^2^ density. Decidualization medium, used for whole differentiation period consisted of: RPMI (phenol free) medium (Corning Incorporated, NY, USA), 2% Charcoal Stripped Fetal Bovine Serum (Gibco, Thermo Scientific, NY, USA) and 50 μg/ml primocin (Sigma Aldrich, MO, USA). Selected decidualization time was 3 and 6 days, cells were cultured under selected conditions of 37°C, 5% CO_2_ in an incubator. During decidualization every other day the medium was replaced with the same fresh medium and filtered through 0.2 μm filter. Undifferentiated EndSCs and MenSCs were used as a control group in later experiments.

### RNA Isolation, cDNA Synthesis and Gene Expression Analysis Using RT-qPCR

RNA from differentiated and undifferentiated EndSCs and MenSCs was isolated by using TRIzol^®^ reagent (Zymo Research, CA, USA), chloroform and isopropanol for RNA precipitation. After this cDNA was synthesized with SensiFAST cDNA Synthesis Kit (Bioline, London, UK) by following manufacturers recommendations. RT-qPCR was performed using SensiFAST SYBR NoRox Kit (Bioline, London, UK) and Rotor-Gene 6000 Real-time Analyzer (Corbett Life Science, QIAGEN, Hilden, Germany) with experimental conditions as follows: 95°C 2 min, 95°C 5 s, X °C (primer melting temperature) 10 s, 72°C 20 s. RT-qPCR was repeated for 40 cycles for all genes and samples. Used primers are listed in the Supplementary Table S1. The acquired data from RT-qPCR was further analyzed using ∆∆Ct method. By using this method every sample was compared to undifferentiated control and normalized using GAPDH gene expression and therefore relative gene expression was calculated.

### Total Protein Isolation and Western Blot Analysis

Proteins from endometrium and menstrual blood stem cells early and late passages were extracted first by using 0.05% trypsin-EDTA (Gibco, Life Technologies, NY, USA) solution on cells. Collected cells (1–1.5 × 10^6^ cells for one sample) are then washed 2 times with ice-cold PBS solution (Genaxxon bioscience, Ulm, Germany) and incubated with benzonase (Merck, Darmstadt, Germany) for 30 min on ice. After this, 1 volume of 2X SDS lysis buffer (62.5 mM Tris, pH 6.8, 100 mM DTT, 2% SDS, 10% glycerol, and traces of bromphenol blue) was added and finally 10 volumes of 1X SDS lysis buffer was mixed into all samples. Then samples were homogenized with a syringe (26 G), incubated for 5 min at 96°C and centrifuged at 20,000 ⨯ g for 15 min, at 4°C. The supernatant was collected and used for SDS-PAGE electrophoresis or stored at −20°C. The proteins were fractionated in 7.5–15% gradient polyacrylamide gels, transferred onto PVDF membrane (Immobilon P; Millipore, Billerica, MA, USA). Western blot analysis was performed using antibodies against cyclin E1 (Merck Millipore, Burlington, MA, JAV), H3K27me3 (Merck Millipore, Burlington, MA, JAV), EZH2 (Thermo Fisher Scientific, Waltham, MA, USA), SUZ12 (Cell Signaling Technology, MA, USA) and p53 (Santa Cruz Biotechnology, Texas, USA). Antibodies against GAPDH (Abcam, Cambridge, UK) were used as a protein loading control. To achieve higher sensitivity secondary horseradish peroxidase-conjugated antibodies against mouse or rabbit antibodies (DAKO, Glostrup, Denmark) were used. Target proteins were enhanced using chemiluminescence detection with Clarity Western ECL Substrate (Bio-Rad Laboratories, CA, USA). Detection was performed using ChemiDocTM XRS + system with Image LabTM Software (Bio-Rad Laboratories, CA, USA). Relative density of target proteins was determined with ImageJ software (NIH, MD, USA) and normalized to the GAPDH loading control.

### Enzyme-Linked Immunosorbent Assay—ELISA

After 6 days of decidualization, the medium from the cells was collected and the concentration of proteins prolactin and IGFBP-1 was measured using ELISA. To determine the concentration of prolactin, DiaMetra Prolactin ELISA Kit and protocol (DiaMetra S.r.l, Italy) was used. For IGFBP-1 concentration determination Mediagnost IGFBP-1 ELISA Kit was used (Mediagnost, Germany). The 96 plate with loaded samples was then measured using Infinite M200 Pro plate reader with i-control 1.5 software (Tecan, Männedorf, Switzerland). After optical density was detected at 450 and 630 nm wavelength, calibration curve was graphed based on the given standards of known concentrations. Then prolactin and IGFBP-1 concentrations (ng/ml) were calculated.

### Statistical Analysis

All experiments were repeated at least 3 times, unless specified otherwise. Statistical analysis was performed using GraphPad Prism (GraphPad Software, USA) software and one-way ANOVA test, followed by Tukey’s multiple pairwise comparison test. Statistical significance is marked as follows: *p ≤ 0.05, **p ≤ 0.01, ***p ≤ 0.001. Data were expressed as mean values ±SDs.

## Results

### Human EndSCs and MenSCs Characterization

**FIGURE 1:**
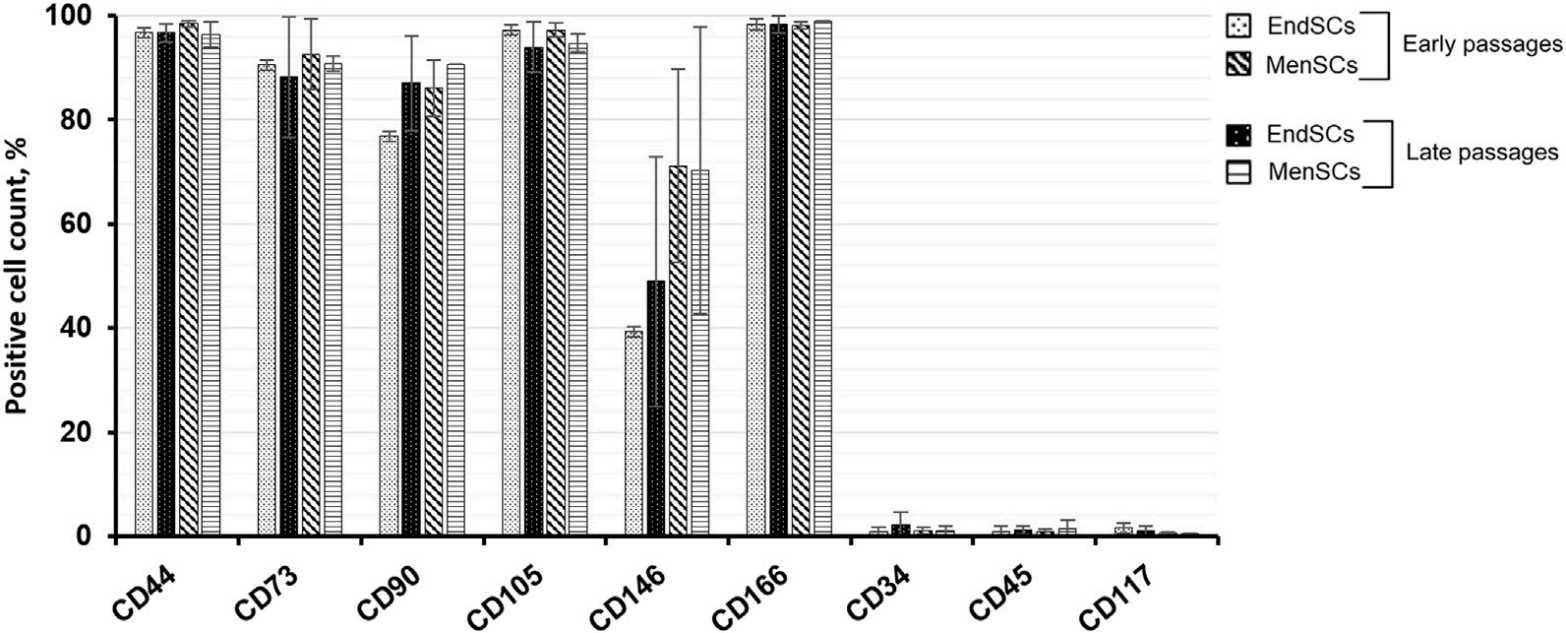
EndSCs and MenSCs cell surface markers analysis. The expression of typical mesenchymal stem cells markers CD44, CD73, CD90, CD105, CD166; hematopoietic progenitor cells and endothelial cells markers CD34, CD45, CD117 and CD146 were detected at early and late passages using flow cytometry. Results are presented as mean ± SD (*n* = 3).

All measured endometrial and menstrual blood stem cells express mesenchymal cell surface markers, such as CD44 (homing cell adhesion molecule), CD73 (5′-nucleotidase), CD90 (thymocyte differentiation antigen 1) and CD105 (endoglin) and at a similar level as identified by flow cytometry (Figure 1). In most of the cases, more than 95% of cell population were marked as strongly positive for these typical mesenchymal stem cell markers. Less than 1% of EndSCs and MenSCs expressed CD34 (hematopoietic progenitor cell antigen), CD45 (leukocyte common antigen) and CD117 (c-kit) surface markers and they were characterized as negative. No significant differences between early and late passages of EndSCs and MenSCs were detected.

While analyzing perivascular marker’s CD146 expression, it was noticed that EndSCs expression was lower than that of MenSCs, although the expression between passages was similar. Both EndSCs and MenSCs expressed activated leukocyte cell adhesion surface marker CD166 at a high level at its expression was maintained at similar level throughout long-term cultivation.

The cells were also characterized by their proliferation rate. During continuous cultivation of EndSCs and MenSCs, the doubling time and viability of these cells was assessed. Examination of EndSCs showed that cell doubling time of early and late passages was very similar and around 2.5–3 ± 0.3 days. The doubling time of MenSCs was approximately 3 ± 0.2 days and did not differ significantly between early and late passages.

### Expression of Stem Cells Pluripotency Markers in Early and Late Passages

The ability of stem cells to maintain its stemness during numerous duplications was evaluated by gene expression of common pluripotency markers: *OCT4, SOX2, NANOG* and *KLF4* using RT-qPCR*.* The appropriate Ct value of every gene and cell line is listed in the Supplementary Table S2. For this experiment as a control group human amniotic fluid mesenchymal stem cells (AF-MSCs) were used since they resemble pluripotent stem cells the most and are the closest to embryonic stem cells that we can use in laboratory setting.

**FIGURE 2:**
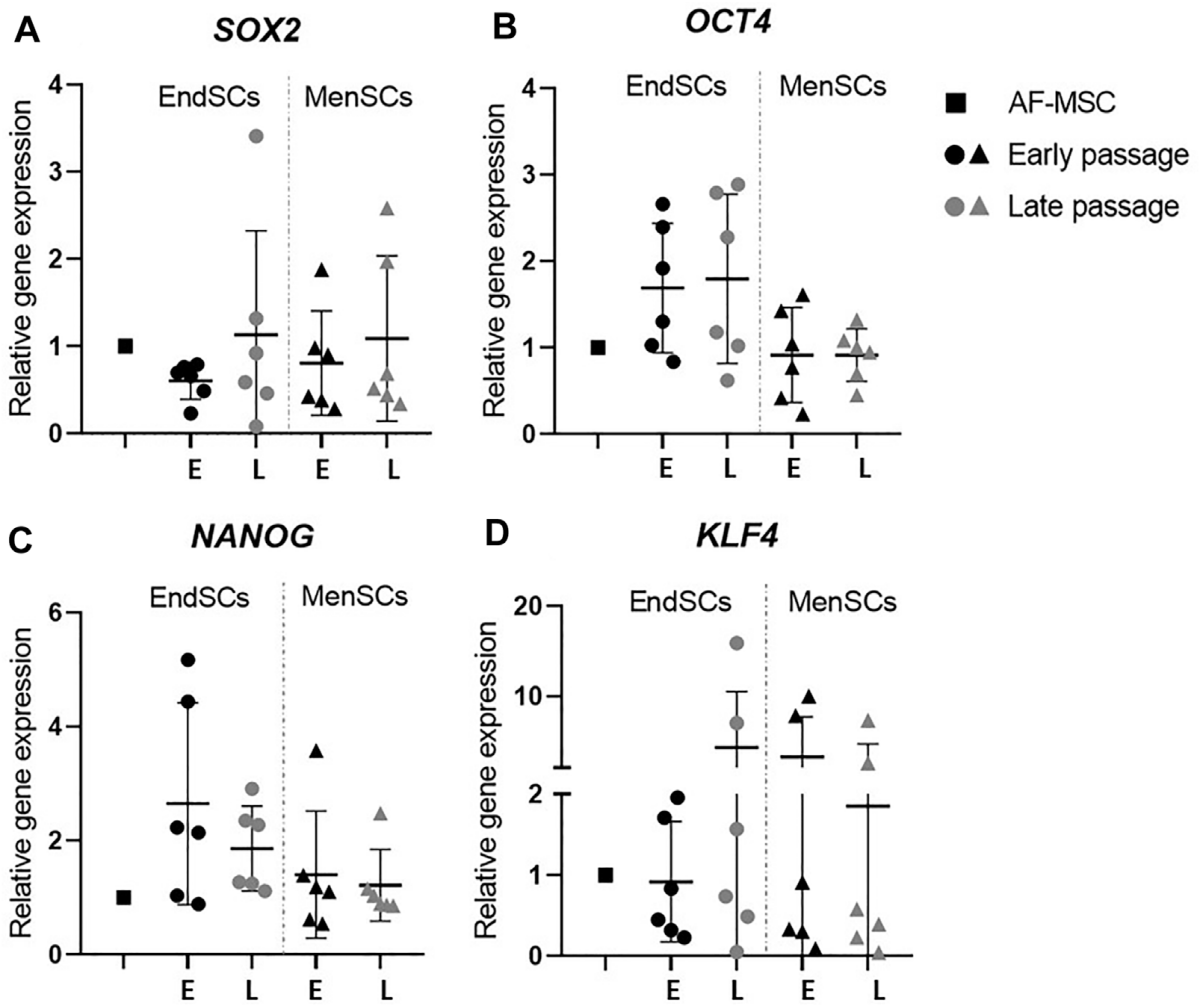
Gene expression analysis of stemness genes in EndSCs and MenSCs. **(A,B,C,D)** relative gene expression of stem cells pluripotency markers *SOX2, OCT4, NANOG* and *KLF4* was detected in early and late passages and measured using RT-qPCR. E—early passage, L—late passage. Relative gene expression was calculated using ΔΔCt method. Data were normalized to GAPDH and presented as mean ± SD (*n* = 6). No statistically significant changes were detected between different passages and cell groups as evaluated by one-way ANOVA.

Relative gene expression values of *SOX2* gene in endometrial and menstrual blood stem cells were very similar in early and late passages, however, the values in most cases were lower than those of a control group—AF-MSCs (Figure 2A). During long-term cultivation of EndSCs and MenSCs *OCT4* and *NANOG* gene expression was consistent, although it was noticed that the expression of these markers was decreased slightly in MenSCs compared to EndSCs, but these changes were not statistically significant (Figures 2B,C). Another mesenchymal stem cells gene-marker *KLF4*, responsible for regulation of stemness and proliferation, showed a similar expression in early as well as late passages in endometrial and menstrual blood stem cells (Figure 2D).

### Expression of Genes Associated With Cell Cycle Regulation in Early and Late Passages

Next six genes (*p53, p21, Rb, CCNA2, CCNE2, CDK2*) associated with cell cycle regulation were analyzed. Sudden upregulation or downregulation of these genes could indicate that cells are reacting to possible negative environmental stressors. For example, upregulation of genes such as *p53, p21, Rb* is associated with cell cycle arrest, on the contrary, the downregulation of *CCNA2, CCNE2, CDK2* genes will lead to decreased proliferative activity.

**FIGURE 3:**
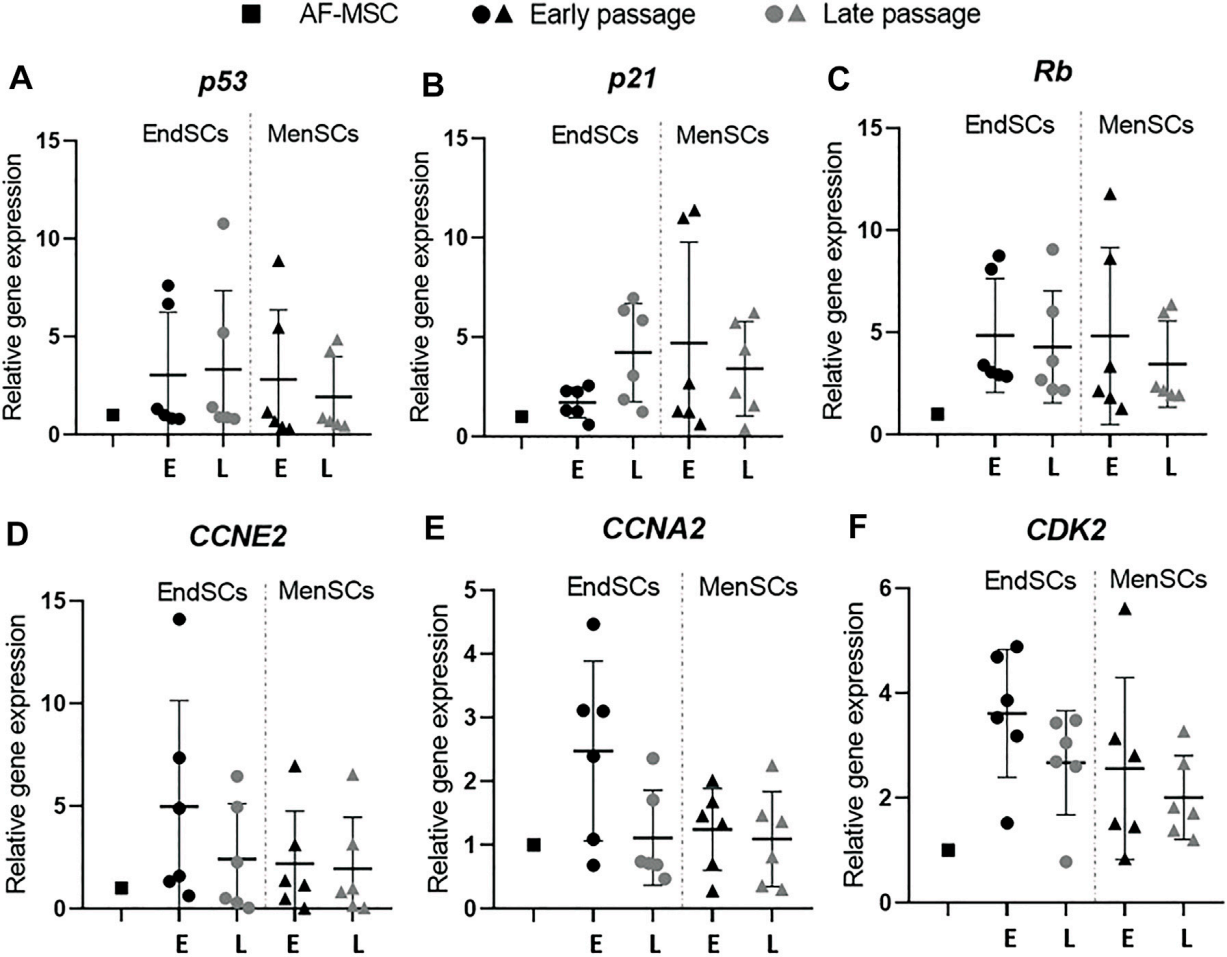
Gene expression analysis of cell cycle regulation associated genes in EndSCs and MenSCs. **(A–F)** relative gene expression of *p53, p21, Rb, CCNA2, CCNE2, CDK2* genes was detected in early and late passages and measured using RT-qPCR. E—early passage, L—late passage. Relative gene expression was calculated using ΔΔCt method. Data were normalized to GAPDH and presented as mean ± SD (*n* = 6). No statistically significant changes were detected between different passages and cell groups as evaluated by one-way ANOVA.

RT-qPCR analysis identified that *p53* gene’s expression in endometrial and menstrual blood stem cells was at a comparable level to control group and did not fluctuate during long-term cultivation (Figure 3A). A relative gene expression of *p21* gene was upregulated in EndSCs and MenSCs in late passages compared to early passages, however, such change was not significant (Figure 3B). The expression profile of *Rb* gene showed a similar tendency as *p53* gene—expression remained at a similar level throughout long-term cultivation, but was slightly higher than in control cells (Figure 3C). By evaluating *CCNE2* gene’s relative expression no significant changes were observed—expression values were at a similar level in early and late passages of EndSCs and MenSCs as well as in control group (Figure 3D). Minimal differences, though, not significant are seen in *CCNA2* and *CDK2* genes expression profiles. Relative gene expression of *CCNA2* and *CDK2* in endometrial mesenchymal stem cells late passages was downregulated compared to early passages (Figures 3E,F). However, menstrual blood stem cells have maintained this expression at a stable position in both early and late passages.

### Expression of Genes Associated With Senescence in Early and Late Passages

**FIGURE 4:**
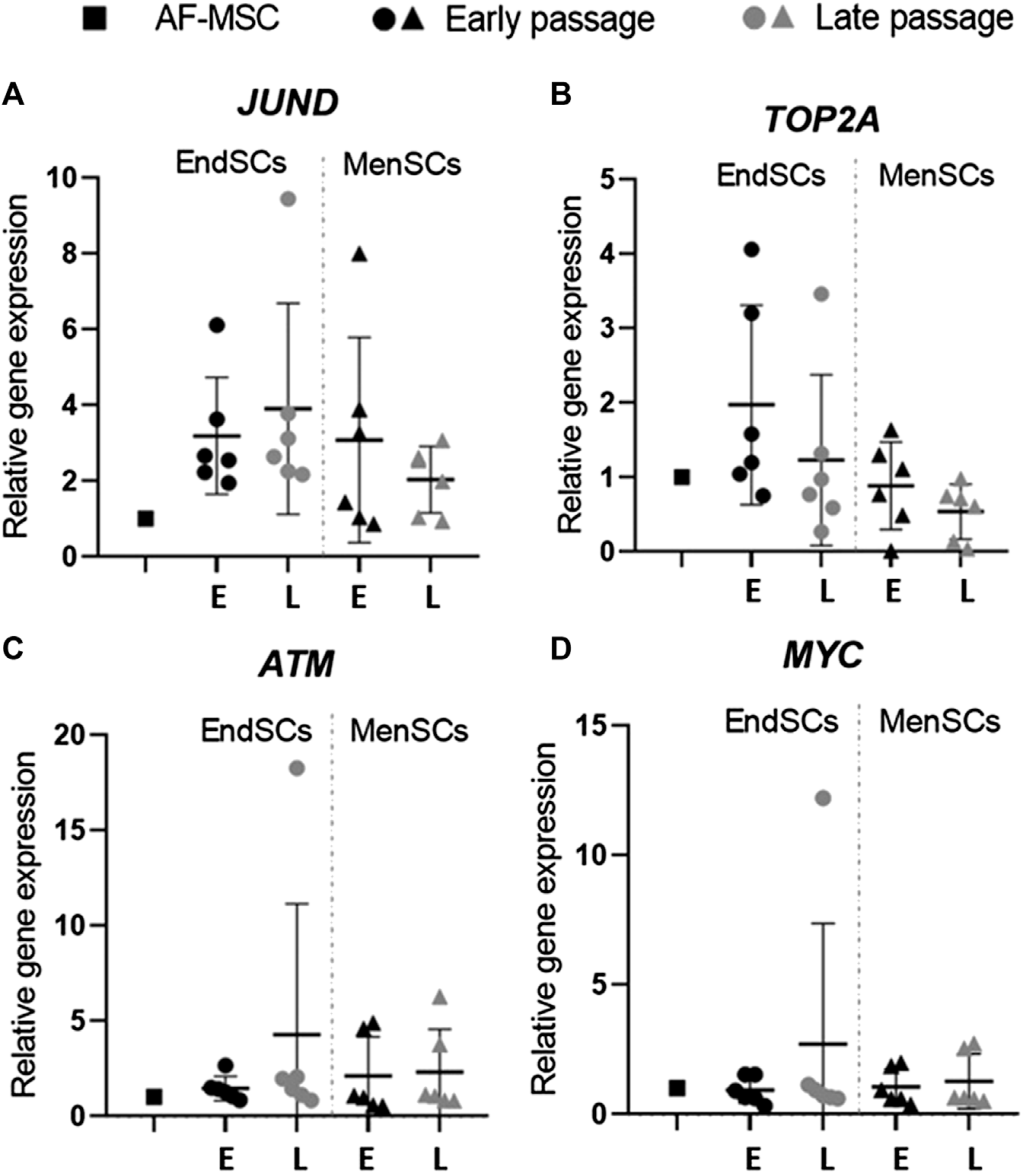
Gene expression analysis of senescence associated genes in EndSCs and MenSCs. **(A–D)** relative gene expression of *JUND, TOP2A, ATM, MYC* genes was detected in early and late passages and measured using RT-qPCR. E—early passage, L—late passage. Relative gene expression was calculated using ΔΔCt method. Data were normalized to GAPDH and presented as mean ± SD (*n* = 6). No statistically significant changes were detected between different passages and cell groups as evaluated by one-way ANOVA.

Since the decrease in proliferation rate can occur not only due to DNA damage responses and other environmental stressors, but also due to the initiation of senescence in cells, it is important to analyze such changes in cells that go through long-term cultivation. In some instances, young cells can experience premature senescence, which slows down their proliferation potency and weakens therapeutic abilities. For this reason, genes *JUND, TOP2A, ATM* and *MYC* that are often related to senescence were examined by RT-qPCR. Prior to gene expression analysis, we carried out β-galactosidase test on cells to determine whether EndSCs and MenSCs exhibit β-galactosidase activity, which is often a characteristic of senescent cells. We performed this test following (Savickiene et al., 2016) protocol on early and late passage endometrium and menstrual blood stem cells. The representative images are provided in Supplementary Figure S1. The results did not demonstrate any significant differences between passages associated with senescence: the morphology remained similar throughout passaging and there was minimal to none β-galactosidase activity. To make sure that gene expression pattern was not altered in EndSCs and MenSC, we then continued to perform gene expression analysis using senescence associated genes. We demonstrated that endometrial stem cells expressed *JUND* gene at a higher level than control group and menstrual blood stem cells, though such increase is not statistically significant (Figure 4A). *JUND* expression in MenSCs did not display significant differences among different passages—it was constant and resembled control group. Relative *TOP2A* gene expression in endometrial stem cells late passages was downregulated in comparison to EndSCs early passaged and became similar to early passages of menstrual blood stem cells (Figure 4B). There was a small decline in the expression levels of *TOP2A* gene in late passages of MenSCs, but overall in both experimental groups this expression remained similar to that of a control group. The *ATM* and *MYC* genes show similar changes in their gene expression profiles. The expression of *ATM* gene in EndSCs and MenSCs was almost identical to that of *MYC* gene (Figures 4C,D). During long-term cultivation senescence marker genes remained similar to or lower than control group, which might indicate that genes responsible for senescence are not yet activated in these cells.

### Changes in Proteins’ Levels Involved in Epigenetic Regulation and Cell Cycle Control During Long-Term Cultivation

**FIGURE 5:**
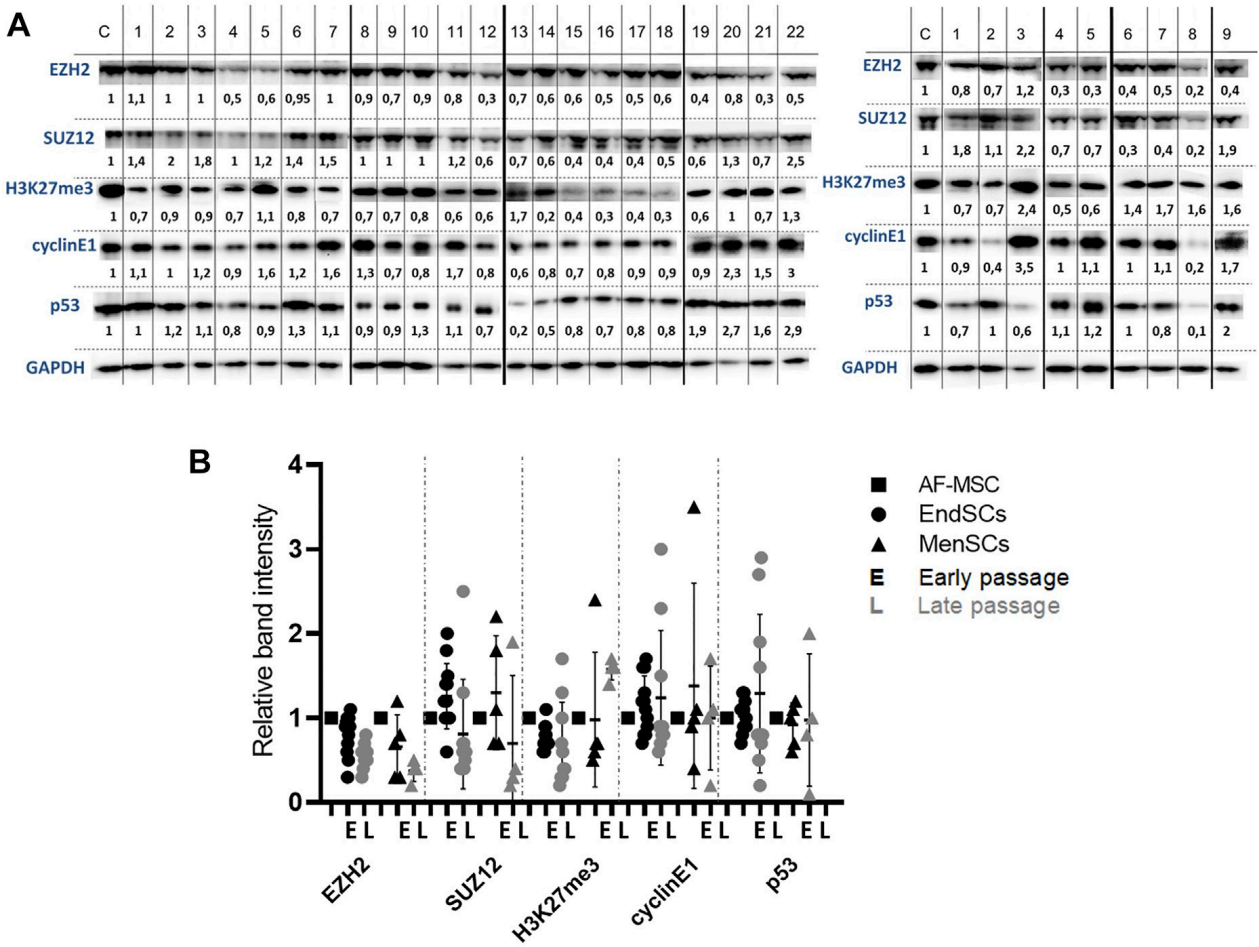
Evaluation of protein level changes in EndSCs and MenSCs. **(A)** C—control cells (AF-MSCs), 1–22 and 1–9—depicts different patients. Left panel shows EndSCs samples, right panel—MenSCs samples. Changes in protein EZH2, SUZ12, H3K27me3, cyclin E1 and p53 levels in early and late passages of EndSCs and MenSCs were determined using Western blot analysis. Relative protein intensity of bands was calculated using ImageJ software and presented under each band. Data were normalized to the GAPDH loading control. **(B)** A graphical representation of detected protein level changes as calculated by ImageJ software and normalized to the GAPDH loading control. Results are presented as mean ± SD (*n* = 6). No statistically significant changes were detected between different passages and cell groups as evaluated by one-way ANOVA.

Further, we explored with epigenetics and cell cycle regulation associated changes at protein expression levels in early and late passages of EndSCs and MenSCs. As demonstrated by Western blot analysis, levels of histone methyltransferases EZH2 (Enhancer of Zeste 2 Polycomb Repressive Complex 2 Subunit) and SUZ12 were downregulated in EndSCs and MenSCs during propagation and became lower than in control group cells (Figure 5A).

These two proteins are members of Polycomb repressive complex 2, which in stem cells is responsible for the maintenance of bivalent state of chromatin. Protein levels of modified histone H3K27me3, which is associated with transcriptional repression and inactive chromatin, remained the same in early and late passages of EndSCs, but were slightly enhanced in MenSCs late passages. Although, this alteration is statistically insignificant, but it may be linked to repression of certain genes. The next assessed protein group represents proteins that are involved in cell cycle control. Both cyclin E1 and p53 proteins’ levels fluctuated depending on a patient, but overall did not show any significant changes and were mostly maintained at a control group level (Figure 5A). Relative band intensity of detected proteins and protein levels were presented by graphical representation (Figure 5B). The assessment of protein levels of EZH2, SUZ12, and H3K27me3 along with gene expression profiles allow us to foresee changes that are happening, when cells duplicate multiple times. This can later be used to determine suitability of such cells to be used in therapy as potential regeneration tool.

### Assessment of Decidualization Initiation in EndSCs and MenSCs

**FIGURE 6:**
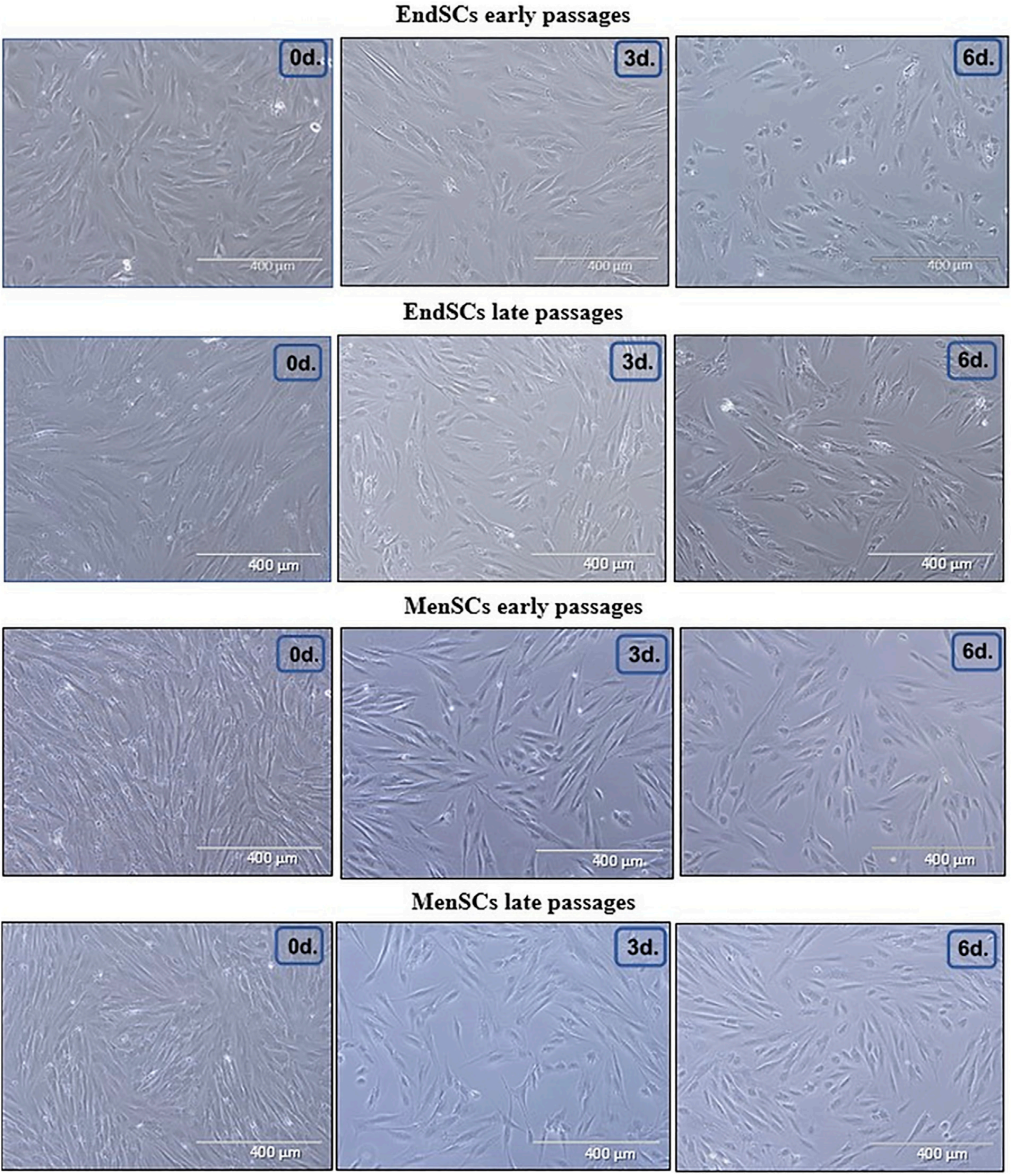
Morphological characterization of EndSCs and MenSCs during decidualization. Morphological alterations were evaluated in early and late passages of EndSCs and MenSCs after 3 and 6 days of decidualization. 0d.—undifferentiated control cells, 3d.—cells after 3 days of decidualization, 6d.—cells after 6 days of decidualization. Scale bar = 400 µm.

**FIGURE 7:**
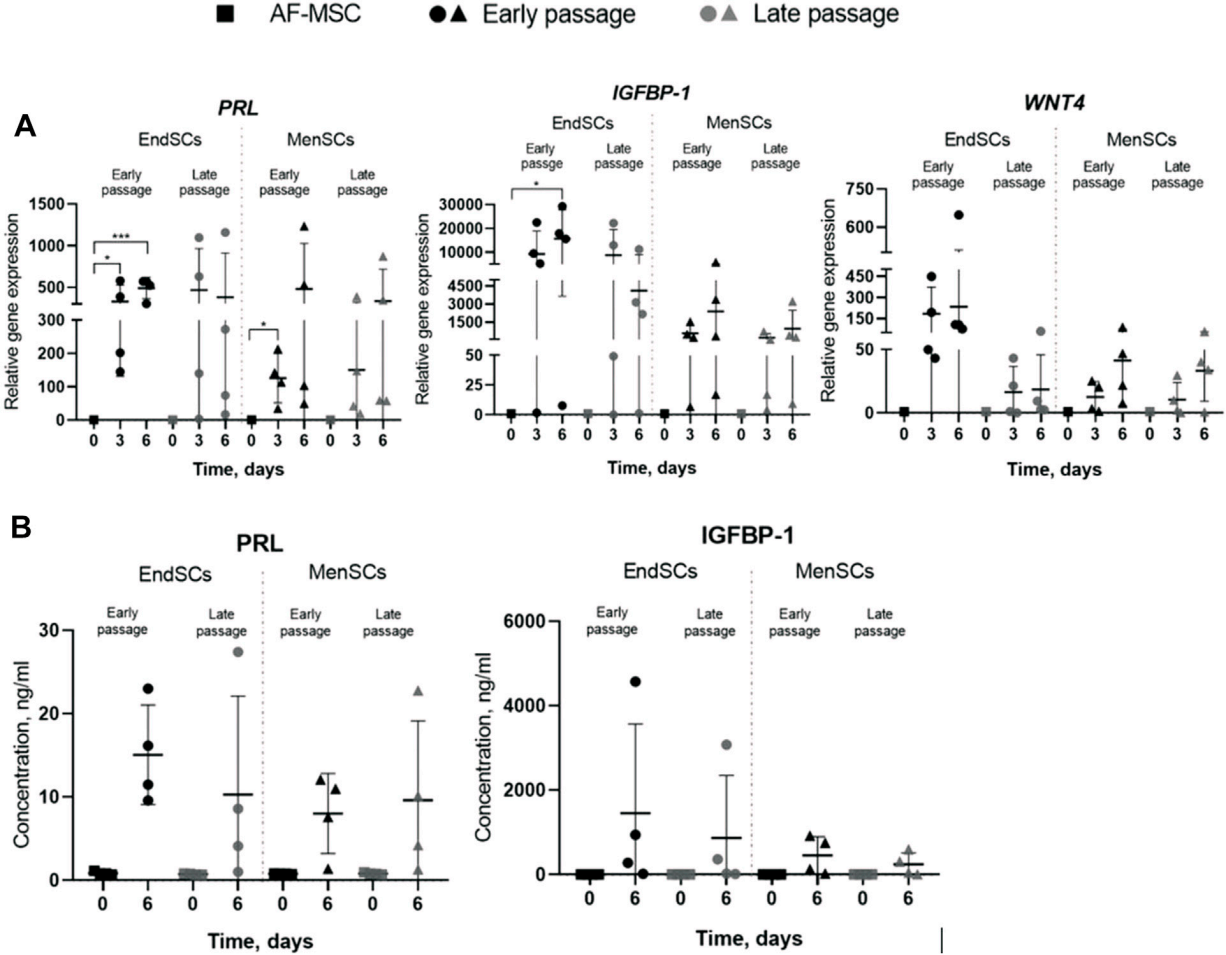
Assessment of decidualization of EndSCs and MenSCs at gene expression level and protein level. 0d.—undifferentiated control cells, 3d.—cells after 3 days of decidualization, 6d.—cells after 6 days of decidualization. **(A)** relative gene expression of decidualization gene markers *PRL, IGBFP-1, WNT4* genes was detected in early and late passages after 3 and 6 days of decidualization induction. Relative gene expression was measured using RT-qPCR and calculated using ΔΔCt method. Data were normalized to GAPDH and presented as mean ± SD (*n* = 6). **(B)** Concentrations of decidualization markers PRL and IGFBP-1 were detected in early and late passages after 6 days of decidualization induction using ELISA. Data represent mean ± SD (*n* = 4). * indicates *p* ≤ 0.05; ** indicates *p* ≤ 0.01; *** indicates *p* ≤ 0.001 compared to undifferentiated control. No statistically significant changes were detected between different passages and cell groups as evaluated by one-way ANOVA.

As mentioned before, decidualization plays a critical role in implantation of blastocyst and eventually pregnancy, for this reason a large focus of this investigation was directed towards the assessment of decidualization during long-term cultivation. The cells were evaluated by a few criteria: morphology, decidualization gene markers’ *PRL, IGFBP-1, WNT4* expression and secreted protein levels. Decidualization was induced by cultivating EndSCs and MenSCs in a medium containing db-cAMP and MPA for 3 and 6 days. For this experiment undifferentiated EndSCs and MenSCs were selected as control groups. EndSCs and MenSCs after 3 and 6 days of decidualization became rounder, flatter and reminiscent of epithelial cells compared to undifferentiated cells (Figure 6). As seen in the pictures, morphological alterations after 6 days were more prominent than after 3 days—more cells changed their phenotype from elongated to round, and most probably longer cultivation in conditioned medium has helped cells to achieve a more similar shape to that of epithelial cells. Moreover, there is no significant difference between early and late passages, which could mean that both groups are evenly capable to change their phenotype based on specific inductors. To further explore the process of decidualization on a molecular level, we evaluated gene expression levels of three main decidualization gene markers: *PRL (prolactin), IGFBP-1 (Insulin-like growth factor-binding protein 1), WNT4 (Wnt Family Member 4).* As determined by RT-qPCR, after decidualization induction relative gene expression of *PRL, IGFBP-1, WNT4* was upregulated in all induced cells (Figure 7A). In endometrial and menstrual blood stem cells the expression of *PRL* gene remained at a similar level throughout propagation and after 3 and 6 days of decidualization. *PRL* expression in MenSCs decreased in comparison to EndSCs, although these changes were minor. EndSCs and MenSCs expressed *IGFBP-1* in a similar manner as *PRL* gene: the expression was higher in EndSCs compared to MenSCs, but these differences were not significant. While analysing *WNT4* gene expression profile it is seen that the expression of this gene was increased the most in early passages of EndSCs (Figure 7A). In all other groups *WNT4* expression was maintained at a similar state during whole period of decidualization. It is important to mention, that longer decidualization of 6 days did not result in better expression of decidualization gene markers. Next, we measured levels of proteins secreted into medium during decidualization. Medium after decidualization induction was collected after 6 days and levels of PRL and IGFBP-1 proteins were assessed by ELISA. Both protein markers increased in comparison to undifferentiated control (Figure 7B). The secreted PRL levels were the highest in early passages of EndSCs, later it decreased slightly and became similar to MenSCs levels. Secreted IGFBP-1 protein levels in endometrial stem cells did not change during long-term cultivation.

Moreover, both EndSCs and MenSCs secreted the IGFBP-1 at a similar level and remained consistent throughout propagation. Overall, our findings suggest that in induced endometrial and menstrual blood stem cells the process of decidualization was initiated and late passages just as well as early passages maintain similar ability to undergo differentiation.

## Discussion

In the present study our aim was to determine if endometrial and menstrual blood stem cells change their properties depending on the period of long-term cultivation. For this purpose, cells were evaluated by mesenchymal stem cell surface markers, the expression of stemness, cell cycle regulation and senescence associated gene markers. Furthermore, our main focus was directed towards epigenetic alterations and changes during decidualization. The assessment of long-term cultivation and the effects that it has on EndSCs and MenSCs is necessary to determine whether these cells could be propagated and potentially used in therapy. Considering broad differentiation potential and self-renewal, stem cells play a big role in regenerative medicine and cell therapy to restore damaged tissues and organs. Human endometrium derived mesenchymal stem cells could be a great source of stem cells therapy in the regenerative medicine for the treatment of reproductive system disorders, especially, unexplained infertility or recurrent pregnancy loss. These cells have the ability to substitute damaged or defective cells, for example, in endometrium (Azizi et al., 2018).

Human endometrium is a dynamic structure that experiences more than 400 regeneration and proliferation cycles during woman’s reproductive age and ensures proper functionality of uterus and reproductive potential (Verdi et al., 2014; Kaingade et al., 2017). In the study we demonstrated that all analyzed EndSCs and MenSCs expressed surface markers CD44, CD73, CD90, CD105. Our results agree with Schwab and Gargett showing that endometrial stem cells were positive for CD44, CD73, CD90 and CD105 stem cells markers (Schwab and Gargett, 2007). We also showed that EndSCs and MenSCs expressed perivascular marker CD146 which corresponds with the same study where the authors identified the co-expression of two perivascular markers CD140b and CD146 on endometrial stem cells. Another big part of our experiments was the assessment of changes happening in cells during propagation. Very similar study was published by Kaingade and colleagues, in which they measured the expression of surface markers in various passages: P1, P3, P5 and P10 (Kaingade et al., 2017). The authors confirmed that all passages, early and late, of EndSCs expressed CD44, CD166, CD117, CD140b, CD146 surface markers at a similar level and were negative for CD34, CD45 and CD106 surface markers. These results corroborated our findings and confirmed the fact that EndSCs and MenSCs maintain mesenchymal stem cells markers expression during long-term cultivation.

Stem cells are characterized not only by surface markers, but also by specific genes related with stemness. *OCT4, SOX2, NANOG, KLF4* genes are responsible for maintaining stemness and undifferentiated state in stem cells. In this study, we determined that EndSCs and MenSCs actively expressed typical stem cells marker genes: *OCT4, SOX2, NANOG, KLF4*. Menstrual blood stem cells expressed *OCT4* and *NANOG* genes throughout 12 passages based on Borlongan and colleagues research (Borlongan et al., 2009). Different study depicted isolated endometrium samples from 35 patients, aged 40 years or younger, tested for stemness genes by RT-qPCR (Xiao et al., 2017). *OCT4, SOX2, NANOG* were expressed by all these samples. Although, the expression levels of stemness gene markers depending on the sample were different, but it still indicates that in EndSCs and MenSCs pluripotency maintenance markers were present. Our findings show that stemness genes maintain the same expression level in early and late passages, which indicates that cells do not lose their ability to proliferate and self-renew even after multiple duplications. These cells retain with mesenchymal stem cells associated profile and, in the future, could be a useful tool for regeneration therapy.

The process of senescence has a big impact on cell vitality and functionality. This is especially important for endometrium-derived stem cells since they have to sustain high proliferative and self-renewal capacity to ensure repeated restoration of endometrial tissue in woman’s uterus. Epigenetic modulators, such as histone deacetylase inhibitors, activate with DNA damage response (DDR) associated proteins ATM and p53, which in turn react to stress signals and accordingly modulate cells’ status (Munro et al., 2004). On a molecular level in senescent cells Rb and p53 signalling pathways are activated and they induce cyclin dependent kinase inhibitors p16 and p21 (Chau and Wang, 2003). The upregulation of these genes and protein levels may indicate about the start of senescence program activation in cells. In the study, we determined that *p53* and *Rb* gene expression in EndSCs is not upregulated, on the other hand, *p21* gene expression is slightly increased in late passages compared to early passages and control group. In addition, by Western blot analysis we determined p53 protein level, which remained at a constant level during propagation. This may imply that p53 signalling pathway is not being activated in analyzed cells and that cell cycle is progressing normally. Cell cycle arrest also happens due to activation of p53/p21/Rb and p16/Rb signalling pathways or sudden decrease of cyclins (CCNE2, CCNE1) and cyclin kinases (CDK2). Upon activation of senescence program, proliferation and cell division is slowed down and senescence associated genes such as *JUND, TOP2A, ATM, MYC* are upregulated. Our study has shown that *p53, Rb* and *ATM* expression in EndSC and MenSC is maintained on a level similar to control group and with progression of passages, levels of these proteins are not increased, thus cells do not switch to programmed senescence and cellular cycle is not being halted. However, there is a single exception, with *p21* gene expression increasing towards later passages, which correlates with study of other researchers (Vassilieva et al., 2018). It was previously demonstrated that cells display increased expression of genes *p16, p53, p21*, while CDK2 and cyclin E levels of proteins gets decreased, with the induction of cellular senescence promoting agent called tert-Butyl hydroperoxide (t-BHP) (Yue et al., 2014). In our experiment we have shown that expression profiles of cell cycle associated genes’ (*p53, CDK2, CCNE2*) did not display any critical cell cycle associated changes, whereas p53 and cyclin E1 protein level is maintained on a normal level during long-term cell cultivation: p53 protein level does not increase in later passages, while cyclin E1 level does not significantly decrease. Therefore, it is possible that EndSC as well as MenSC maintain active cell cycle. In 2013, Wang et al. carried out a study in which they were analysing what effect different agents have on cellular division, proliferation and health. They have demonstrated the significance of genes *CCNA2, CCNE2* and *p53* in arresting cell cycle (Wang et al., 2013). In the study, gene expression and protein level analysis revealed that *CCNA2, CCNE2, CDK1* gene expression was decreased both *in vivo* and *in vitro* which resulted in the arrest of cell cycle in S phase (*CCNA2, CCNE2*) and G2/M phase (*CDK1*). Such trend was also confirmed by performing Western blot analysis. While these results do not correlate with our research results, in this case it is a positive outcome, proving the absence of cell cycle arrest in EndSC and MenSC.

In order for endometrial stem cells to be used in regenerative therapy, they must demonstrate genetic and epigenetic stability. The major concern with stem cells and their applicability in clinical practices is the correct expansion of these cells *in vitro* under controlled conditions. Stem cells must duplicate for multiple times to produce sufficient number of cells, whilst avoiding the disruption of genetic and epigenetic stability, because only stable cell populations can be used for stem cell based therapy (Sharma and Bhonde, 2020). Moreover, studies exploring characteristics of mesenchymal stem cells during long-term cultivation showed that long-term culture could affect morphology, functional properties and overall quality of cells (Wagner, 2012; Galderisi and Giordano, 2014). As mentioned before, tissue regeneration especially in endometrium is one of the most important processes defying healthy female’s cycle and reproduction probability. Regeneration facilitates changes in cells’ transcriptional programs as well as in signalling pathways to ensure correct reconstruction of damaged organs. This is achieved by epigenetic regulation, which alters chromatin structure through DNA modifications and histone post-translational modifications (acetylation, methylation and phosphorylation). An important part of regeneration process is the preservation of tissue-specific cell characteristics throughout cell’s lifetime by regulation of self-renewal, proliferation and differentiation (Chiacchiera et al., 2020). Specific gene expression programs that are regulated by epigenetic modifications, which modify chromatin’s state and genes’ accessibility to certain transcription factors, determine these processes. For example, by silencing with stemness associated genes, differentiation program will be activated, which will cause stem cells to alter their gene expression pattern and activate specialized genes for appropriate cell fate determination (Ozkul and Galderisi, 2016). We measured protein levels of epigenetic regulatory markers EZH2, SUZ12 and H3K27me3 linked to histone methylation and modification in endometrial and menstrual stem cells during propagation. EZH2 and SUZ12 are both members of Polycomb Repressive Complex 2 (PRC2), which catalyzes the di-methylation of histone H3 at lysine 27 (H3K27me3). Polycomb group (PcG) protein complexes are essential for regulation of cell cycle control, cell fate decisions and stem cell differentiation. Furthermore, PcG complexes are involved in post-translational modifications of histones, which in turn result in transcriptionally repressive chromatin (Morey and Helin, 2010). We observed that EZH2 and SUZ12 protein levels decreased slightly in EndSCs and MenSCs during long-term cultivation and in most cases were lower than in early passage cells and in control group. Although, this decrease is not statistically significant, these results may show that EndSCs and MenSCs undergo some changes during long-term cultivation. A study carried out by Bracken et al. (2007) implicated that EZH2 protein levels tend to be downregulated in senescent cells, so that differentiation program could not be activated (Bracken et al., 2007). On the other hand, several cancers are associated with EZH2 overexpression (Bentivegna et al., 2013). Similar results were displayed in Jung and colleagues (2010) study, in which the authors examined epigenetic changes in umbilical cord blood-derived mesenchymal stem cells in early and late passages. Immunoblot analysis suggested that levels of SUZ12 and EZH2 proteins are decreased in late passages compared to early passages (Jung et al., 2010). This downregulation was linked to replicative senescence and loss of self-renewal as well as proliferative capacity. In addition, the analysis of DNA methyltransferases (DNMT) activity in fetal placental mesenchymal stem cells during propagation revealed some changes in DNMTs expression profiles. It was revealed that in late passages (P8) DNMTs were downregulated compared to early passages (P3) as determined by RT-qPCR (Zhu et al., 2015). Although, histone methylation at lysine site can result in the activation or repression of genes, H3K27me3 is typically associated with repressed chromatin (Katoh et al., 2018). In this study H3K27me3 mark remained at a similar level in EndSCs early and late passages, but was upregulated in late passages of MenSCs. There is a lack of available information about histone methylation patterns in stem cells during long-term propagation, but it is clear that chromatin state experiences some changes during cell cycle progression (Sharma and Bhonde, 2020). Moreover, methylation sites at key regulatory gene promoters represent different states (active, repressed, poised) and may influence cell fate determination and developmental commitment (Mikkelsen et al., 2007).

Our work has shown a successful decidualization of EndSCs and MenSCs to epithelial progenitor cells *in vitro*. Decidualization was induced by using db-cAMP and MPA agents and confirmed by evaluating phenotypical changes, decidualization gene markers *PRL, IGFBP-1*, *WNT4* expression and IGFBP-1, PRL protein level changes. Some studies have shown, that by cultivating cells in medium containing 0.5 mM db-cAMP for 4 days, successful decidualization is induced in endometrial mesenchymal stem cells (Tamura et al., 2012). There are many publications, in which decidualization is successfully induced using different cAMP forms and combinations with MPA (Aghajanova et al., 2009; Logan et al., 2012; Katoh et al., 2018; Xiong et al., 2020). The assessment of cells’ morphology, gene expression and protein levels are the main methods by which decidualization progress is often evaluated. Our results show that after 3 and 6 days of decidualization induction the morphology of EndSCs and MenSCs changed—the cells acquired similar phenotype to epithelial cells. The expression of *IGFBP-1, PRL* and *WNT4* genes increased and secreted proteins IGFBP-1, PRL were upregulated as measured by ELISA. Similar experiments were carried out by Saleh and colleagues, as determined by RT-qPCR the expression of *IGFBP-1* and *PRL* genes was upregulated compared to undifferentiated control cells (Saleh et al., 2011). In the same study the authors evaluated protein levels using ELISA and Western blot methods. The results were corresponding with our findings—both IGFBP-1 and PRL protein levels were upregulated. It was previously found out that WNT4 is responsible for regulation of decidualization process in the endometrial stromal cells during embryo implantation (Franco et al., 2011). Since this gene is associated with decidualization, we determined that it was increased in EndSCs and MenSCs, especially in early passages of EndSCs. On the contrary, in late passages of MenSCs *WNT4* expression is decreased slightly, although in most cases it is higher than in undifferentiated cells. All these results combined confirm that EndSCs and MenSCs are able to differentiate to epithelial progenitor cells under controlled conditions. Our tested minimal period of decidualization was 3 days and it yielded similar results in morphology, gene expression and protein levels as after 6 days. The achieved successful decidualization might indicate that if injected to damaged uterus these cells could possibly be used to promote reparation of damaged tissue by initiating proliferation of adjacent stem cells.

## Conclusion

In this study we have shown that EndSCs and MenSCs while being cultivated for a long period of time maintain their stem cell abilities and are a promising tool for future therapy to treat reproductive system disorders. We also concluded that changes in protein levels associated with epigenetic regulation do not differ significantly in early and late passages. Overall, endometrial stem cells and menstrual stem cells in most cases show very similar characteristics and both are able to undergo decidualization. Although, more research is necessary to determine the potential use of these stem cells for cell-based therapy, our results may demonstrate that stem cells could potentially be used to treat reproductive system disorders in the future, especially, unexplained infertility, in order to achieve successful embryo implantation and pregnancy development.

## Data Availability

The data presented in this study are available on request from the corresponding author.
