# Supplementary material for: Decidualization Potency and Epigenetic Changes in Human Endometrial Origin Stem Cells During Propagation
Source: Front Cell Dev Biol. 2021 Nov 19;9:765265. doi: 10.3389/fcell.2021.765265 (PMC8640123; doi:10.3389/fcell.2021.765265)
Supplement: Supplementary file 1 [file DataSheet2.PDF]

Supplementary Table S2. Threshold cycle (Ct) values of *SOX2*, *OCT4*, *NANOG*, *KLF4* genes calculated by RT-qPCR. The table represents Ct values – the number of cycles necessary to determine the level of gene expression. For every cell line (AF-MSC, EndSC, MenSC), there is a representative Ct value listed according to a specific gene (n=6).

**Genes** / **Cell lines**	***SOX2***	***OCT4***	***NANOG***	***KLF4***
	Threshold cycle (Ct) values generated in RT-qPCR			
AF-MSC	30	24	25	25
EndSC	31	24 ± 1	23-24	26 ± 1
MenSC	30 ± 1	24 ± 1	24-25	27 ± 1
