# Supplementary material for: Decidualization Potency and Epigenetic Changes in Human Endometrial Origin Stem Cells During Propagation
Source: Front Cell Dev Biol. 2021 Nov 19;9:765265. doi: 10.3389/fcell.2021.765265 (PMC8640123; doi:10.3389/fcell.2021.765265)
Supplement: Supplementary file 2 [file Table1.DOCX]

**Supplementary Table S1.** List of primers used for gene expression in RT-qPCR.

| **Gene** | **Primer Sequence (5‘-3‘), F – forward, R – reverse** | **Product Size (bp)** |
| --- | --- | --- |
| ***GAPDH*** | F 5‘GTGAACCATGAGAAGTATGACAAC  R 5‘-CATGAGTCCTTCCACGATACC | 123 |
| ***OCT4*** | F 5‘-CGAGAAGGATGTGGTCCGAG  R 5‘-CAGAGGAAAGGACACTGGTC | 136 |
| ***SOX2*** | F 5‘-TGGACAGTTACGCGCACAT  R 5‘-CGAGTAGGACATGCTGTAGGT | 215 |
| ***NANOG*** | F 5‘-AGATGCCTCACACGGAGACT  R 5‘-GTTTGCCTTTGGGACTGGTG | 96 |
| ***KLF4*** | F 5‘-CACATTAATGAGGCAGCCACC  R 5‘-AAGTCGCTTCATGTGGGAGAG | 169 |
| ***p53*** | F 5‘-TAACAGTTCCTGCATGGGCGGC R 5‘-AGGACAGGCACAAACACGCACC | 121 |
| ***p21*** | F 5‘-GGCAGACCAGCATGACAGATT R 5‘-GCGGATTAGGGCTTCCTCT | 73 |
| ***Rb*** | F 5‘-GCAGTATGCTTCCACCAGGC R 5‘-AAGGGCTTCGAGGAATGTGAG | 62 |
| ***CCNA2*** | F 5‘-AACTTCAGCTTGTGGGCACT R 5‘-AAACTCTGCTACTTCTGGGGG | 80 |
| ***CCNE2*** | F 5‘-GATGGAACTCATTATATTAAAGGCTT  R 5‘-AGGAGCATCTTTAAGAGCATCAACTT | 81 |
| ***CDK2*** | F 5‘-TGTCAAGCTGCTGGATGTCA  R 5‘-CAGTGAGAGCAGAGGCATCC | 78 |
| ***JUND*** | F 5‘-CGCCTGGAAGAGAAAGTGAA  R 5‘-GTTGACGTGGCTGAGGACTT | 117 |
| ***TOP2A*** | F 5‘-ATTCCCAAACTCGATGATGC R 5‘-CCCCATATTTGTCTCTCCCA | 136 |
| ***MYC*** | F 5‘AATGAAAAGGCCCCCAAGGTAGTTAT  R 5‘-GTCGTTTCCGCAACAAGTCCTCTTC | 112 |
| ***ATM*** | F 5‘-CTCTGAGTGGCAGCTGGAAGA  R 5‘-TTTAGGCTGGGATTGTTCGCT | 129 |
| ***PRL*** | F 5‘-TGACCCTTCGAGACCTGTTTG  R 5‘-CTTGCTCCTTGTCTTCGGG | 177 |
| ***IGFBP1*** | F 5‘-TTTTACCTGCCAAACTGCAACA R 5‘-CCCATTCCAAGGGTAGACGC | 108 |
| ***WNT4*** | F 5‘-AGGAGGAGACGTGCGAGAAA R 5‘-CGAGTCCATGACTTCCAGGT | 83 |
