# Supplementary figures and images for: Decidualization Potency and Epigenetic Changes in Human Endometrial Origin Stem Cells During Propagation
Source: Front Cell Dev Biol. 2021 Nov 19;9:765265. doi: 10.3389/fcell.2021.765265 (PMC8640123; doi:10.3389/fcell.2021.765265)

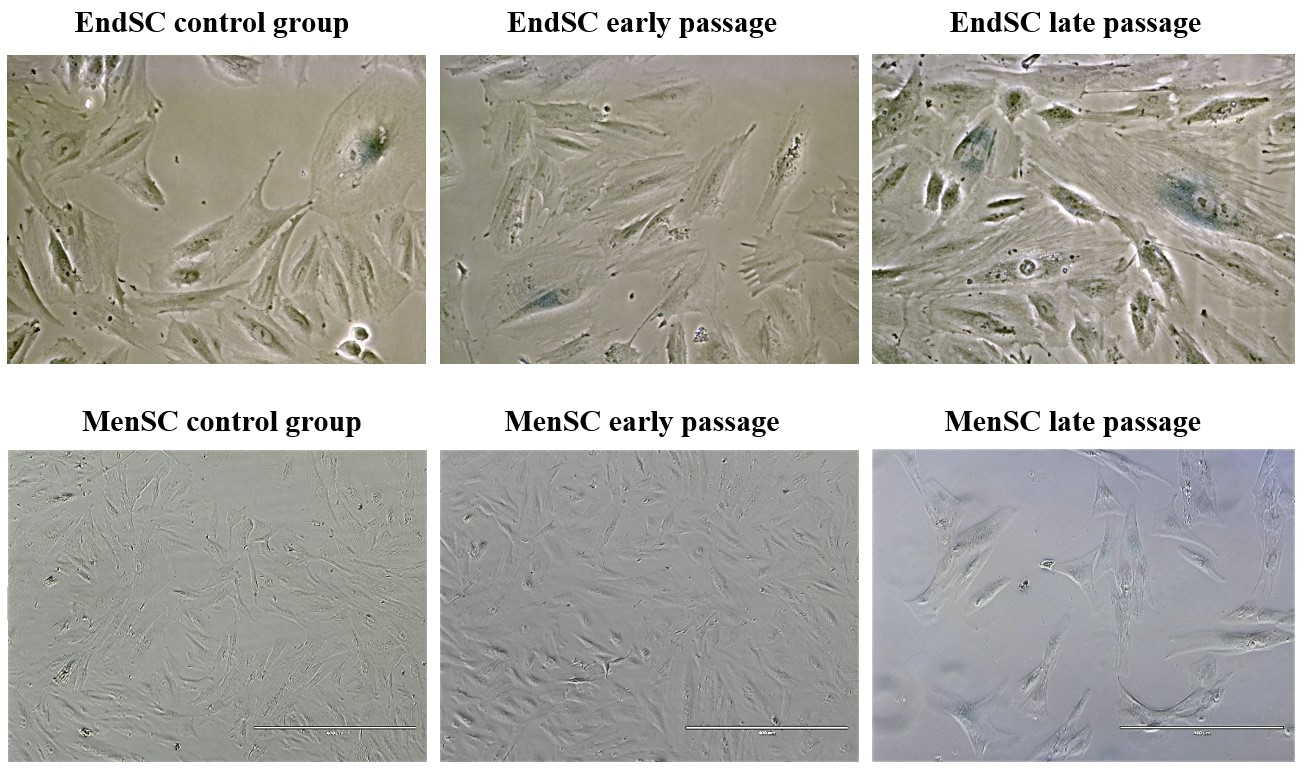

Supplement: Supplementary file 3 [file Image1.TIF]
