# Supplementary material for: Decidualization Potency and Epigenetic Changes in Human Endometrial Origin Stem Cells During Propagation
Source: Front Cell Dev Biol. 2021 Nov 19;9:765265. doi: 10.3389/fcell.2021.765265 (PMC8640123; doi:10.3389/fcell.2021.765265)
Supplement: Supplementary file 4 [file DataSheet1.PDF]

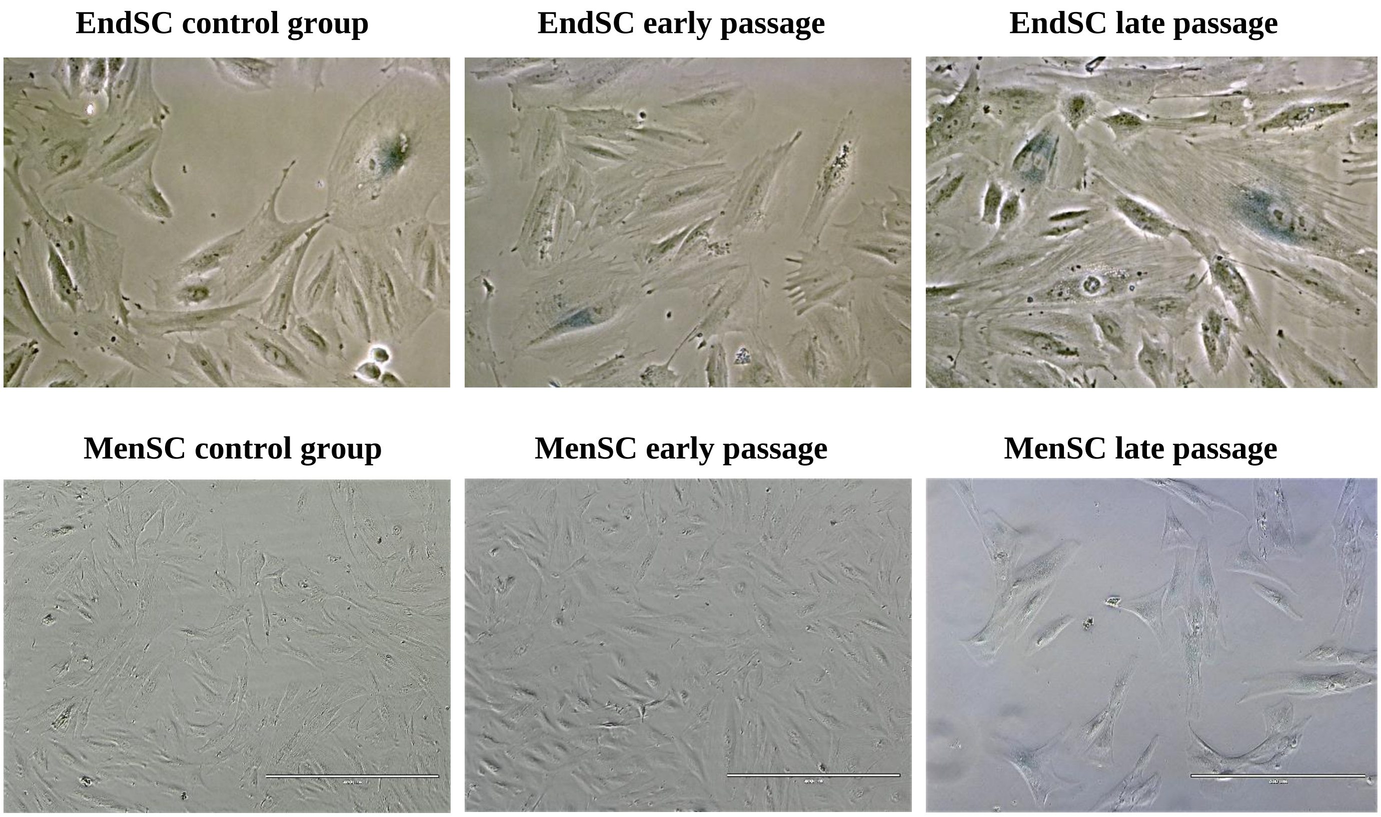

Supplementary Figure S1. Representative images of β-galactosidase staining test on EndSCs and MenSCs. Early and late passage cells were stained with β-galactosidase for the assessment of senescence in cells during passaging. Color blue indicates β-galactosidase activity, which is characteristic of senescent cells. Scale bars = 100 μm and 400 μm.
